# Effects of Table Grape Cultivars on Fruit Quality and Aroma Components

**DOI:** 10.3390/foods12183371

**Published:** 2023-09-08

**Authors:** Wan-Ni Wang, Yun-Hui Qian, Ruo-Han Liu, Tao Liang, Yin-Ting Ding, Xue-Lei Xu, Shan Huang, Yu-Lin Fang, Yan-Lun Ju

**Affiliations:** 1 College of Enology, Northwest A&F University, Yangling 712100, China; wangwanni@nwafu.edu.cn (W.-N.W.); qianyunhui@nwafu.edu.cn (Y.-H.Q.); 2018013767@nwafu.edu.cn (R.-H.L.); 18684142780@163.com (T.L.); dyting@nwafu.edu.cn (Y.-T.D.); xlxu@nwafu.edu.cn (X.-L.X.); fangyulin@nwsuaf.edu.cn (Y.-L.F.); 2 Yangling Rural Economic Management Service Station, Yangling 712100, China; 13720581718@163.com; 3 Heyang Viti-viniculture Station, Northwest A&F University, Yangling 712100, China

**Keywords:** vitis, nutritional quality, flavor compounds, sensory evaluation

## Abstract

The basic physical and chemical qualities, nutrition, aroma components, and sensory evaluation of 17 varieties of table grapes were studied. The quality evaluation system of different table grape varieties was preliminarily determined. Our results show that the soluble solid content in *Ruby Seedless* was 21.17%, which was higher than that of other varieties. The black varieties *Aishenmeigui* and *Sweet Sapphire* had the highest total phenol content. *Aishenmeigui* had high levels of tannin and vitamin C. In addition, the aroma contents in *Meixiangbao*, *Ruby Seedless*, and *Shine-Muscat* were higher than those in other varieties. *Manicure Finger* and *Ruby Seedless* had higher levels of C6 compounds. Moreover, the “Kyoho” series of grape *Meixiangbao*, *Sunmmer Black*, *Jumeigui*, *Hutai 8 hao*, and *Black Beet* were high in ester content, while Muscat varieties, including *Zaoheibao*, *Aishenmeigui*, *Jumeigui*, and *Shine-Muscat* were rich in terpene substances. *Ruby Seedless*, *Shine-Muscat*, and *Heibaladuo* had higher comprehensive scores in sensory evaluation. Hence, the comprehensive quality of *Shine-Muscat*, *Ruby Seedless*, and *Aishenmeigui* was better. These results may serve as references for determining the quality differences between table grape varieties.

## 1. Introduction

Grape is a deciduous vine of the genus *Vitis* labrusca belonging to the Vitaceae family, and its size, shape, skin color, flesh color, flavor, and aroma vary from species to species. Food and Agriculture Organization statistics show that approximately 75,866 square kilometers of agricultural land are used for grape production, and table grapes account for approximately 27% of global grape production [1]. Although wine consumption in China has increased over the past decade, the grape industry is still dominated by table grapes, accounting for 80% of total grape production [2]. At the same time, people consume grapes worldwide owing to the rich nutrition, variety, unique fruit shape, and other characteristics of grapes.

Numerous studies have shown that daily intake of fruits and vegetables can effectively prevent chronic diseases, such as cardiovascular disease, cancer, and diabetes, due to the presence of fiber, minerals, vitamins (vitamins C and E), and phytochemical compounds (including phenolic acids, flavonoids, and anthocyanins) [3]. In addition, studies have shown that grapes contain a variety of vitamins, carotenoids, and polyphenols, which are an important source of health-promoting compounds for humans [4,5,6]. Common intermediate phenolic compounds in grapes include anthocyanins, stilbenes, flavan-3-ol, and tannins [7]. Anthocyanins have certain antioxidant activities preventing diseases, including cardiovascular diseases and cancer [8]. Proanthocyanidins, also known as concentrated tannins, are found in berry skins and seeds and are important for improving wine taste and stability [9]. However, high levels of tannins are associated with a high degree of astringency, which is considered a negative characteristic of food grapes [10]. In addition, grapes are sources of vitamin C, which is generally considered the most important vitamin in human nutrition [11].

Although grapes are an important source of health-promoting compounds, they are consumed primarily for their sweetness, juiciness, and aroma. Aroma is one of the important factors affecting grape quality. For example, consumers are usually attracted to the rose fragrance of the Sunshine Rose grape. Studies have shown that terpenes, C13 isoprene, methoxypyrazine, C6-alcohol, aldehydes, esters, and mercaptans are among the most important compounds that contribute to the aroma of grapes [12]. Among these compounds, terpenes and esters provide floral and fruity characteristics to grapes, while alcohols and C6 aldehydes contribute to a herbaceous flavor [13]. Table grapes are classified into groups depending on the type of aroma [14]. For example, table grapes can be classified into Muscat, strawberry, and fox aromas; among them, monoterpenes are the main compounds in muscat grape varieties [15]. Moreover, recent studies have shown that monoterpenes have antifungal, antibacterial, antioxidant, anticancer, and antispasmodic effects [5,16]. Several epidemiological studies have also suggested the potential of terpenes in preventing and treating breast, skin, lung, colon, and prostate cancers [17]. Several factors, such as variety, tree age, rootstock, and maturity, affect the aroma of grapes [18]. Currently, there are few studies on varietal quality differences, especially on varietal aroma characteristics, which need to be further studied.

Thus, in this study, the basic physical and chemical indices, nutritional indices, aroma components, and sensory quality of 17 different table grape varieties were analyzed. Some high-quality table grape varieties with a sweet and sour flavor, juicy flesh, and rich texture were selected. The results lay a foundation for determining the quality differences among varieties.

## 2. Materials and Methods

### 2.1. Samples

The grapes were obtained in 2021 at a commercial vineyard in Yangling, Shaanxi, China (33°17′ N, 107°04′ E) (Table S1). The annual precipitation in Yangling is 635.1–663.9 mm with an average annual temperature of 12.9 °C. Ten vines were selected for each variety of grape. Three clusters were randomly selected from each vine, 10 berries from each cluster, for 300 grape berries. To reduce experimental errors, two trees at the beginning and the end of each row were avoided. Healthy fruits uniform in size were used in the experiment. All grape samples were immediately frozen with liquid nitrogen and stored in a −80 °C refrigerator. All samples were triplicated.

### 2.2. Physical–Chemical Analysis

A single grape berry was weighed on a FA2018N electronic balance (Jinghua Science and Technology Instrument Co. Ltd., Shanghai, China) after washing with distilled water and drying with filter paper. The pH was measured with a PHS-3C lightning magnetic pH meter (Precision Science Instruments Co. Ltd., Shanghai, China). Brix values were measured with a hand-held digital Atago PAL-1 m (Atago Co. Ltd., Tokyo, Japan). According to OIV (2012), the titratable acid was determined through NaOH titrimetric method (using tartaric acid as a meter).

### 2.3. Nutritional Quality Analysis

The content of total anthocyanins in red grape berries was determined using the pH differential method with dimethyl anthocyanin (mg/kg) [13]. The flavonoid content was determined using the sodium nitrite-aluminum nitrate method [19]. The total phenol content was determined using the Folin-Ciocalte method [20]. The content of vitamin C in fruits was determined using the 2,6-dichloroindophenol method [21]. The tannin content was determined using the Folin–Denis method [22].

### 2.4. Aroma Compounds Determined Using Gas Chromatography–Mass Spectrometry

Headspace solid-phase microextraction was used to extract aroma compounds from grapes [23]. Free aroma compounds were extracted from berries using previously reported methods [24,25]. The grape was ground into 50 g homogenate in liquid nitrogen (during which 5 g PVPP was added). After soaking for 2.5 h, the supernatant was separated through centrifugation at 8000 rpm for 10 min. Approximately 1.00 g of NaCl and 5 mL of grape juice were added to the sample bottle, and 10 μL of internal standard substance 4-methyl-2-amyl alcohol (2.02 mg/L) was added to the mixture. All samples were uniformly vibrated with a magnetically heated agitator at 40 °C set to 30 min. An activated extraction head (50/30 ΜM DVB/Carboxen/PDMS, Supelco, Bellefonte, PA, USA) was inserted into the sample bottle air layer for 35 min at 40 °C for extraction. The extraction head was placed in a gas chromatograph (GC) inlet for 5 min to analyze the grape aroma according to the method established in our laboratory.

The GC was operated under the following conditions: the carrier gas was helium (He), and the flow rate was 1 mL/min. The procedure of rising column temperature was as follows: 40 °C was increased to 160 °C at 4 °C/min for 3 min, then to 230 °C at 7 °C/min for 8 min, and finally to 250 °C (inlet), and 1 μL of the injection volume.

Mass spectrometer conditions: electron ionization source (EI), electron source voltage (70 eV), filament flow (0.20 mA), ion source temperature (230 °C), detector voltage (350 V), mass spectrum scanning range (33–450 amu), and scanning frequency (1 Hz).

Qualitative and quantitative methods: The quality spectra obtained by GC-MS analysis were compared with the NIST14.L spectrum library of computer for qualitative analysis of aroma compounds. The compounds were identified using the methods of standard retention time comparison, literature retention index comparison and aroma characteristics comparison. The method of the internal standard–standard curve was used for semi-quantitative analysis with 4-methyl-2-pentanol as the internal standard.

### 2.5. Sensory Evaluation Analysis

The sensory evaluation group consists of 15 professionally trained wine students, including 7 boys and 8 girls, aged between 22 and 28. The sensory evaluation of the wine was evaluated from four aspects: appearance analysis (clarity, chroma, and hue), flavor analysis (purity, concentration, and elegance), texture analysis (purity, concentration, balance, persistence, and flavor characteristic quality), and overall evaluation, with a total score of 100 points (Table S2).

### 2.6. Statistical Analysis

The data were analyzed using SPSS 23.0, the sensory characteristic scores were analyzed using an independent-sample t-test, and the other indices were analyzed using a one-way analysis of variance. The difference was significant (*p* < 0.05), and the values were expressed as mean ± standard error. Partial least squares discriminant analysis (PLS-DA) of aroma compounds was performed with Simca 14.1 software, and GraphPad PRISM 8.0.2 software was used for mapping.

## 3. Results and Discussion

### 3.1. Basic Physical and Chemical Quality of Different Table Grape Varieties

There were significant differences in fruit weight per grain among different varieties. As shown in Table 1, the average berry weights of *Aishenmeigui* and *Jumeigui* were 10.87 g and 10.37 g, respectively, higher than those of other varieties. The average single berry weight of *Shaoxing 1 hao* was only 3.76 g, which was lower than that of other varieties. In previously reported studies, the average berry weight of *Hutai 8 hao* was 10.40 g [26]. However, the average berry weight of *Hutai 8 hao* was 9.28 g in this study, slightly lower than the normal average berry weight.

The maturity of grapes was preliminarily determined using the pH value and the content of soluble solids. Table 1 shows significant differences in the pH value of all grape varieties. *Aishenmeigui* has the highest pH of 4.45. The pH values of *Meixiangbao* and *Italia* were 3.04 and 3.13, respectively, lower than those of other varieties. Apart from *Sweet Sapphire* and *Zitianwuhe*, the other 15 varieties contained more than 16% of the soluble solid. The soluble solid content in *Ruby Seedless* was 21.17%, and that of *Zitianwuhe* was the lowest, only 15.71%. The titratable acid contents in 17 grape cultivars were between 6 and 9 g/L. Among the grape cultivars, the titratable acid contents in *Zaoheibao* and *Italy* were 8.55 g/L and 8.63 g/L, respectively, higher than those of other varieties. The titratable acid content of *Meixiangbao* was the lowest (6.77 g/L).

### 3.2. Nutritional Quality of Different Table Grape Varieties

**Figure 1:**
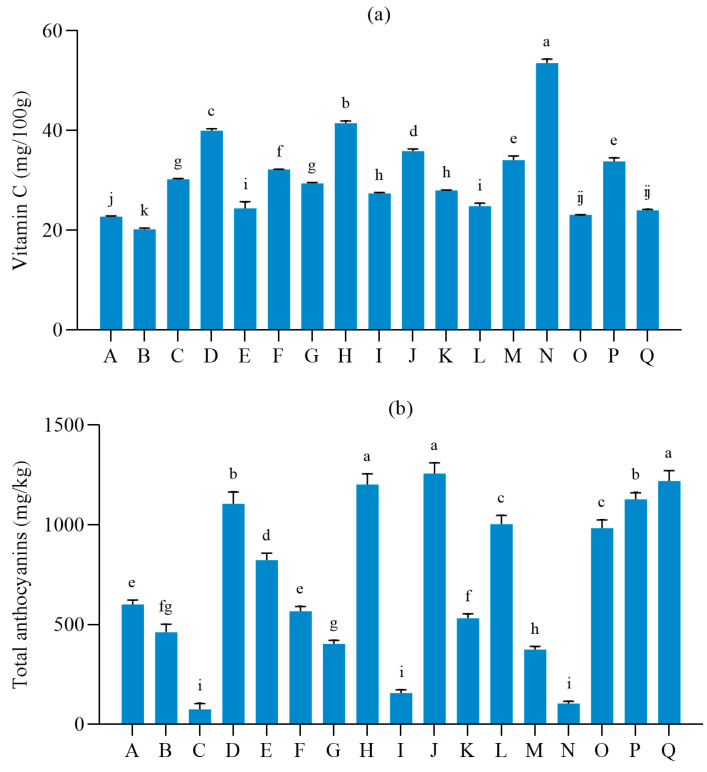

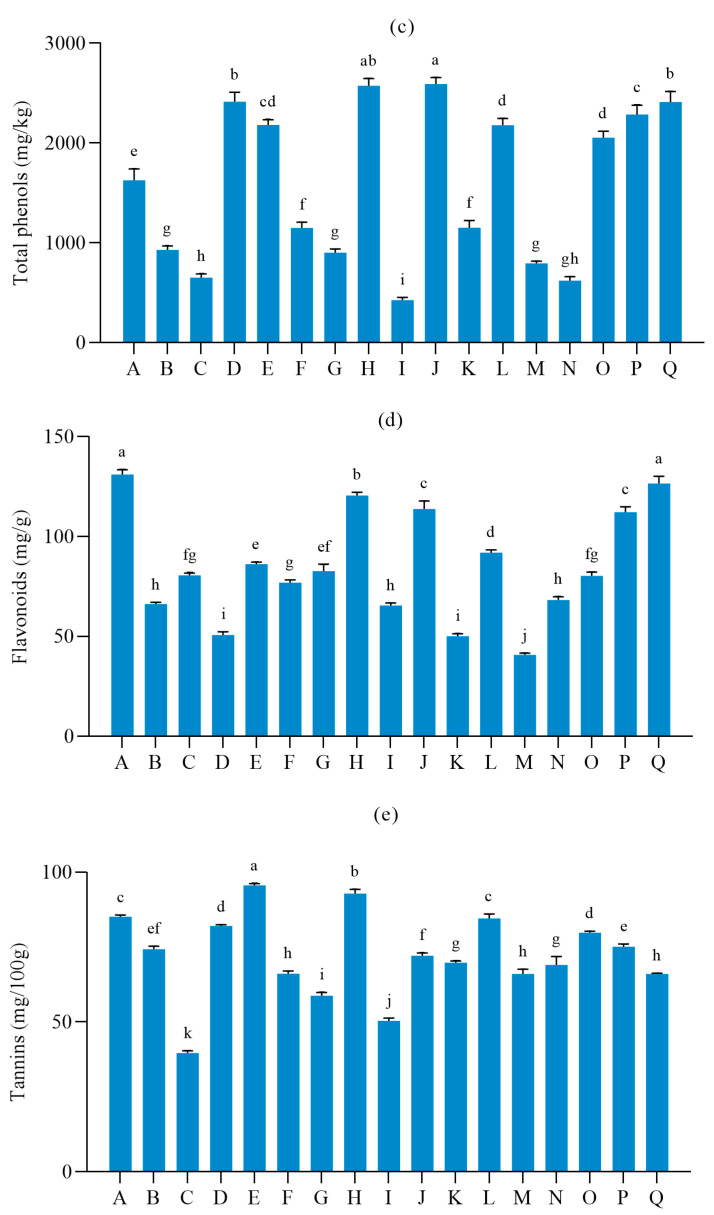
Nutritional quality analysis of different table grapes. (**a**), Vitamin C; (**b**), Total anthocyanins; (**c**), Total phenols; (**d**), Flavonoids; (**e**), Tannins. Lower-case letters indicate significant (*p* < 0.05).

The nutritional quality analysis of different table grape varieties is shown in Figure 1. Vitamin C is an important nutrient and antioxidant in grapes that can eliminate free radicals and reduce oxidative stress. There was a significant difference in vitamin C content among different table grape varieties (Figure 1a). The grape varieties with high vitamin C content were *Shine-Muscat* (53.51 mg/100 g) and *Aishenmeigui* (41.45 mg/100 g). The grape varieties with vitamin C content ranging from 30 to 40 mg/100 g were *Sunmmer Black*, *Zaoheibao*, *Sweet Sapphire*, *Jumeigui*, and *black beet*. The other 10 varieties contain vitamin C ranging from 20 to 30 mg/100 g.

The content of total anthocyanin depends on the color of the grape peel. The content of total anthocyanin was higher in the grape varieties with purple-black color. Figure 1b shows a significant difference in total anthocyanin content between light-colored grape and dark-colored grape varieties. The anthocyanin contents in *Sunmmer Black*, *Aishenmeigui*, *Sweet Sapphire*, and *Black Beet* were each more than 1000 mg/kg. The total anthocyanin contents of *Italia* and *Shine-Muscat* were 74.96 mg/kg and 104.89 mg/kg, respectively, lower than those of other varieties. Although *Zhengyanwuhe* is a red variety, the total anthocyanin content in this variety was only 156.53 mg/kg due to its small fruit grains and thin pericarp.

Total phenol is an important organic active substance and secondary metabolite in grapes, affecting the flavor and taste. Figure 1c shows no significant difference between *Aishenmeigui* and *Sweet Sapphire* total phenol contents. However, the total phenol contents in *Aishenmeigui* and *Sweet Sapphire* were higher than that in other varieties. The total phenol contents in *Italia*, *Zhengyanwuhe*, *Jumeigui*, and *Shine-Muscat* were below 800 mg/kg, while *Zhengyanwuhe* was approximately 426.63 mg/kg, which was lower than other varieties.

Flavonoids can enhance the ability of anti-oxidation and free radical scavenging and have a certain bacteriostatic effect [27]. There were significant differences in flavonoid content among different table grape varieties (Figure 1d). The flavonoid content in *Zitianwuhe* was 126.58 mg/g, while the content of vitamin C was 23.96 mg/100 g. The contents of vitamin C and flavonoids in *Aishenmeigui* were 41.45 mg/100 g and 120.60 mg/g, respectively, higher than those in other varieties.

Tannin is a natural antioxidant and preservative that imparts a certain bitterness to grapes. The tannin contents of *Shaoxing 1 hao* and *Aishenmeigui* were 95.59 mg/100 g and 92.85 mg/100 g, respectively (Figure 1e). The tannin content in seedless early-maturing variety *Italia* was lower than that of other varieties, approximately 39.59 mg/100 g.

### 3.3. Aroma Profiles of Different Table Grape Varieties

The aromatic substances were abundant in grapes. Table 2 shows 81 aroma compounds that were detected in 17 grape cultivars, including seven C6, twenty esters, thirty terpenes, eight alcohols, three acids, nine aldehydes, and three species of C13 isoprene.

The C6 compound, also known as the green component, is an unsaturated fat enzymatic breakdown product and is the essential aroma component of the 17 grape varieties [28]. The contents of C6 compounds in *Italia*, *Manicure Finger*, and *Ruby Seedless* were higher than 2000 μg/L. The content of (E)-2-hexenal was the highest, followed by hexanal, hexanol, and (Z)-2-hexenol, while the content of 3-hexenol and 3-hexenal was lower. Levels of (E)-2-hexenal in all varieties were higher than levels of 3-hexenal, which is consistent with the findings of Aubert et al. [29]. This result illustrates that, in most plants, compounds with a (3Z)-aldehydes structure are rapidly isomerized by (3Z, 2E)-aldehydes isomerase to form the (2E)-aldehydes [30].

The alcohols detected in this study included heptanol, octanol, nonanol, benzyl alcohol, phenylethyl alcohol, 1-octen-3-ol, and 2-ethyl hexanol. The proportion of alcohols in total aroma content was not high, and the content of alcohols in all varieties did not exceed 30 μg/L. Phenylethyl alcohol and 2-ethyl hexanol were found in high concentrations in the 17 grapes, particularly in *Meixiangbao*, *Zaoheibao*, and *Sweet Sapphire*. However, the alcohol had a high threshold of odor inactivation and did not produce the expected floral aroma. The esters detected in this study were ethyl acetate, ethyl butyrate, ethyl heptanoate, ethyl pentanoate, ethyl 2-methylbutyrate, ethyl 3-methylbutanoate, and ethyl hexanoate. The content of ethyl acetate was the highest, and the ethyl acetate had a considerable celery flavor. However, the deactivation threshold was approximately 4700 μg/L, without a significant effect on the overall aroma of the grape. The activity threshold of ethyl 2-methylbutyrate and ethyl 3-methylbutanoate was less than 1 μg/L, and the fruity aroma can be expressed by both of them. Ethyl 2-methylbutyrate and ethyl 3-methylbutanoate contribute significantly to the aromas of *Hutai 8 hao*, *Heibaladuo*, *Sunmmer Black*, *Ruby Seedless*, and *black beet* varieties. Among them, aldehydes and esters were the main volatile components of *Hutai 8 hao*, which is consistent with Yao et al. [31]. Most of the organic acids in grapes are produced through the long-term respiration of the green plants before the color change of the berries, and most of them are found in the peel and seeds. In this study, *Sunmmer Black*, *Jumeigui*, *Hutai 8 hao*, and *Black beet* belonging to the *Kyoho* grapevine series were used due to their advantages including large fruit, bright color, and disease resistance. These cultivars occupy the largest acreage in China. In addition, esters were detected in the four cultivars of the Kyoho grape series, consistent with the results of Wu et al. [14]. In the experiment, only three types of organic acids were detected: hexanoic acid, nonanoic acid, and 2-hexanoic acid. Although the detected organic acids produced unpleasant odors, such as putrefaction and perspiration, they contributed little to the overall aroma.

The terpene aroma compounds measured in this study were the most abundant among all the aroma compounds. Our findings show that the terpenes are the characteristic compounds of the four varieties of Muscat grape, consistent with the findings of Mateo et al. [15]. Terpene forms the basis of the classification of grape cultivars [14]. The contents of terpenes in *Zaoheibao* and *Manicure Finger* were more than 1000 μg/L. In addition, the terpene contents in *Heibaladuo*, *Aishenmeigui*, *Zhengyanwuhe*, *Ruby Seedless*, *Ruiduwuheyi*, *Jumeigui*, and *Shine-Muscat* were between 800 and 1000 μg/L. The difference in aroma between different grape varieties can be attributed to genetic variation in aroma biosynthesis genes. For example, an allelic variant of 1-deoxy-d-xylose-5 phosphate synthase and a terpenoid biosynthesis gene can cause the accumulation of terpenes in the Muscat grape [32]. Cis-(trans-) rose oxide, eugenol, linalool, and citronellol had a low threshold of odor inactivation, which contributed to the overall aroma characteristics of the grape. The main terpene compounds were citronellol, neral, linalool, and α-terpineol, followed by Geranial cis-Linalool oxide, neral, and (Z)-Limonene. The content of cis-(trans-) rose oxide, 4-terpinene, β-terpinene, and hotrienol were lower; because of their very low odor thresholds, linalool and geraniol are generally considered to be the two major flavor contributors to Muscat [15]. Recent studies have shown that monoterpenes play a role in the prevention and treatment of various diseases, including cancer [5]. In particular, linalool is an antioxidant, anti-inflammatory, and cardiovascular stimulant [5,33], and geraniol can inhibit the growth of HEPG2 human hepatoma cells [17,34].

C13-isoprene is a carotenoid aromatic substance. Due to the distinct carotenoid species, regional characteristics, and extremely low threshold of odor inactivation, it plays an important role in the characteristic aroma of a wine. The C13 isoprene detected in this study included β-damascenone, β-Ionone, and geranylacetone. The content of C13 isoprene was low in the total content of all aroma components (lower than 1 μg/L), and its distribution was more uniform in all varieties. C13 isoprene compounds were detected in all varieties except *Ruiduwuheyi*. The β-damascenone and β-Ionone had distinct sweet fruit and floral aromas, and their deactivation thresholds were less than 0.005 μg/L. Therefore, β-damascenone and β-Ionone contributed significantly to the overall aroma characteristics of the grape.

### 3.4. Sensory Evaluation of Different Table Grape Varieties

**Figure 2:**
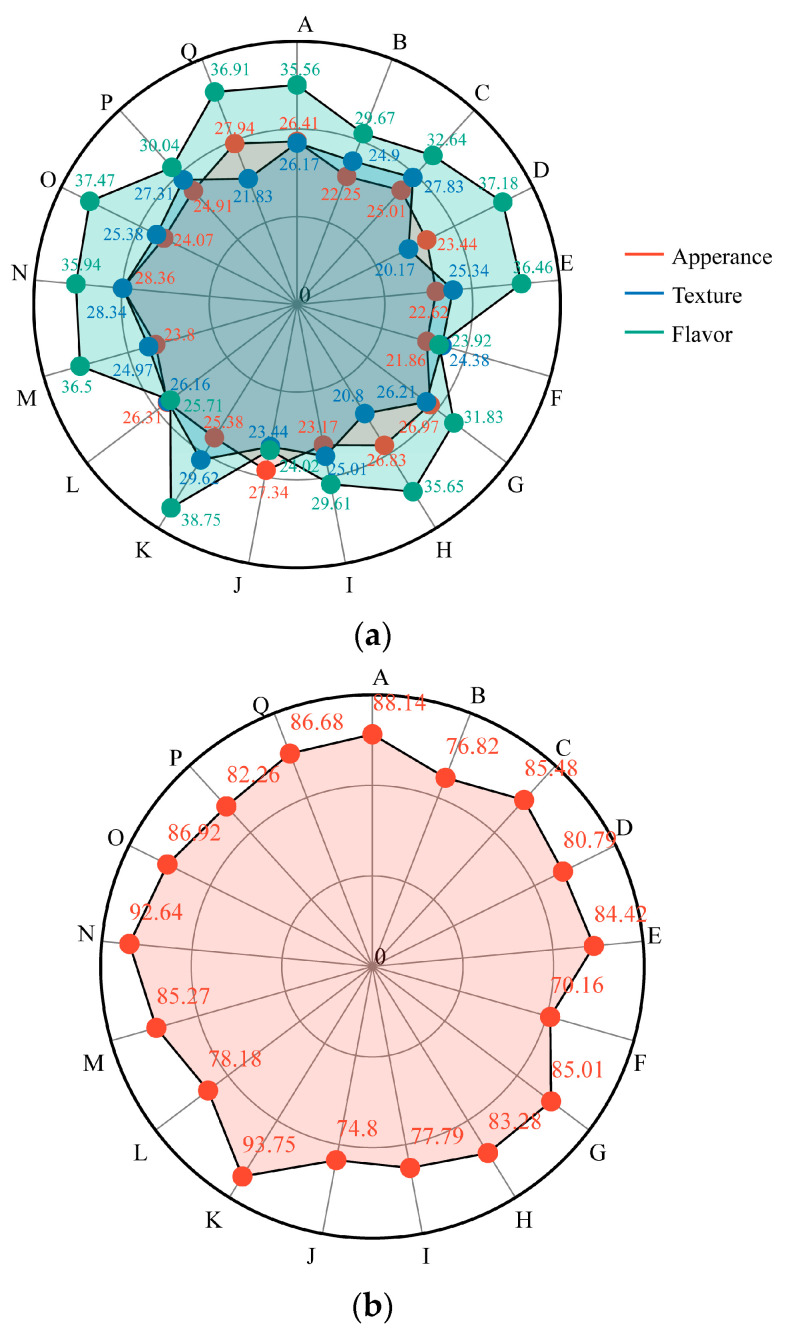
Sensory evaluation scores of different table grape varieties on appearance, texture and flavor (**a**). The comprehensive score of sensory evaluation of different table grape varieties (**b**).

Sensory analysis is a series of observations, analyses, and descriptions of grapes performed through human senses [35]. To examine aroma characteristics and evaluate the comprehensive quality of the grape, 15 wine professionals scored the appearance according to the shape, size, color, and weight of the grape. The texture parameters were scored according to the degree of softness and hardness of grape pulp, the abundance of juice and water, and the brittleness of grape pulp. The flavor parameters were scored according to the coordination of sweet and sour, characteristic aroma, and pure flavor. The sensory evaluation scores and comprehensive scores of different table grape varieties are shown in Figure 2. In appearance, the score of *Shine-Muscat* (28.36 points) was higher than that of other varieties. The appearance scores of the other varieties had no significant difference. In terms of texture, the experts preferred the crispy grape varieties. *Ruby Seedless*, *Shine-Muscat*, *Italia*, *black beet*, and *Heibaladuo* had higher texture scores than other varieties, among which *Ruby Seedless* scored the highest (29.62), while *Sunmmer Black*, with its soft fruit, scored the lowest (20.17). Varieties with a score of more than 35 on the flavor level were *Heibaladuo*, *Sunmmer Black*, *Ruby Seedless*, *Shaoxing 1 hao*, *A*, *Jumeigui*, *Hutai 8 hao*, and *Shine-Muscat*. The flavor scores of *Meixiangbao*, *Zhengyanwuhe*, and *Ruiduwuheyi* were below 30 points, and the varieties had bad flavor and no considerable aroma. *Ruby Seedless* and *Shine-Muscat* scored a combined score of more than 90, higher than the other varieties, and are popular for their rich aroma, crispy texture, and sweet and sour taste. *Sweet Sapphire* tasted sour after ripening, was crisp but astringent, and had a comprehensive score of only 74.81 points.

### 3.5. Principal Component Analysis (PCA) and PLS-DA of Different Table Grape Varieties

**Figure 3:**
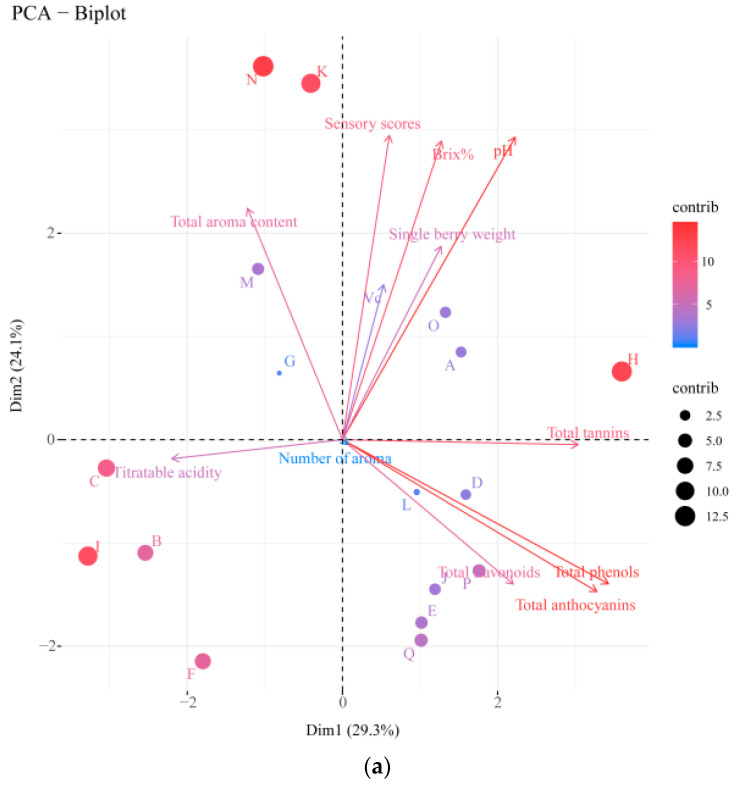

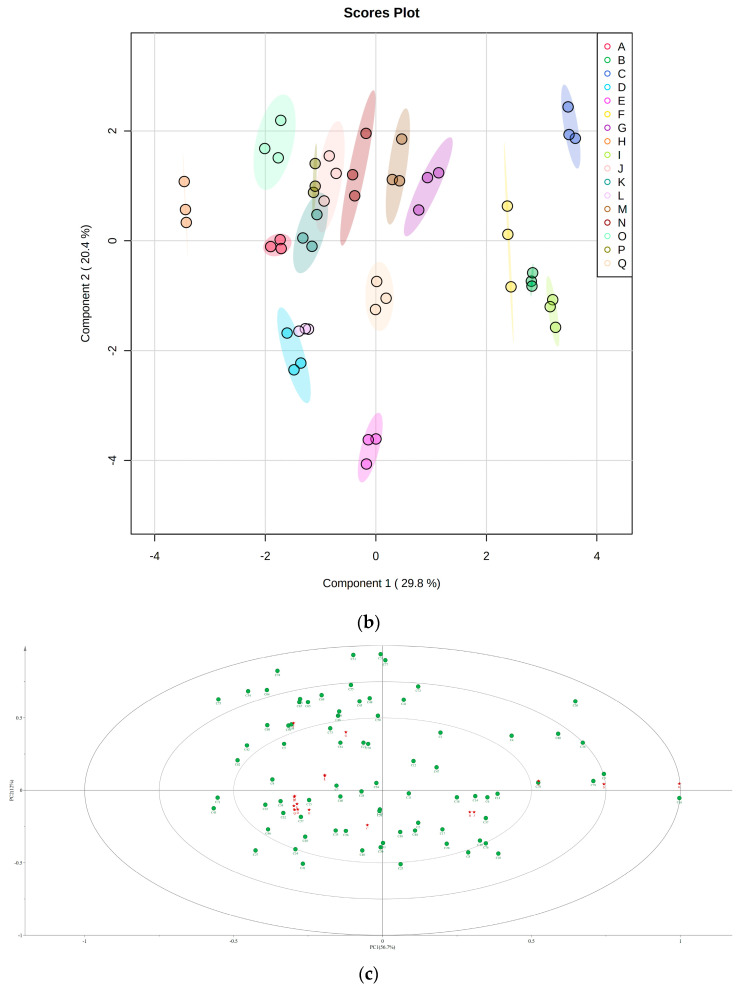
Principal component analysis and partial least squares discriminant analysis of different table grape varieties. Biplots of aroma (**c**) and quality index (**a**) of different table grape varieties; (**b**) Partial least squares discriminant analysis (PLS-DA). The circles represent aroma compounds, and the pentagram represents grape varieties.

In the comprehensive evaluation of the quality of table grapes, the appearance, flavor, texture, and other factors of the fruit should be considered, as well as the nutritional value of the fruit, such as soluble solids and titratable acids, to ensure the comprehensive evaluation of information. The PCA is a multivariable data analysis technique for dimensionality reduction and showing relationships/correlations between variables and samples [36,37]. In this study, 11 main indices that can reflect fruit quality were selected for a comprehensive evaluation of grapes using PCA, and the results were objective. The first two principal components explained 53.4% of the original variables, and varieties C, B, and I were more affected by acids. Varieties M and G had higher contents of aroma components, which could be classified into one group, variety A had higher contents of vitamin C, variety H had higher contents of tannin and C6 compounds, and variety I had higher contents of vitamin C than other varieties, which were close to the principal component axis (Figure 3a).

Biplots (score plots combined with loading plots, Figure 3c) of the PCA showed that 68.7% of the variance was explained by 80 different aroma components, with PC1 and PC2 accounting for 56.7% and 12% of the variance, respectively. Except for varieties C, G, L, F, A, N, and K, the other varieties were not well differentiated. However, the L, G, F, A, N, and K varieties were located at the positive end of the second principal component. The other varieties were located at the negative end of the second principal component, and the difference in positions was obvious.

To explain the experimental results more comprehensively, PLS-DA was used to analyze the obtained data. As shown in Figure 3b, the first two principal components (PC1 and PC2) accounted for 50.2% of all variables. The results showed that 17 varieties could be clearly distinguished from each other. The results were consistent with PCA (Figure 3a), further validating the objectivity and rationality of the data analysis.

### 3.6. Cluster Analysis of Different Table Grape Varieties

**Figure 4:**
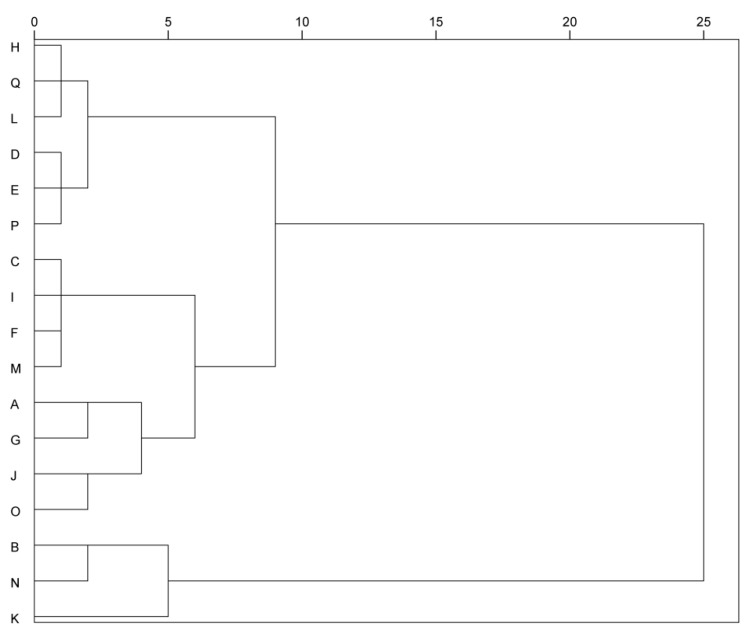
Genealogy of 17 different table grape varieties for cluster analysis.

Cluster analysis can solve the problem of classification when there is more than one index and these indices have great correlation. The classification indices selected in this study included pH, titratable acid, soluble solid, single berry weight, total anthocyanin, flavonoid, total phenol, tannin, vitamin C, total sensory score, the total content of aroma substances and aroma types, a total of 15 species. As shown in Figure 4, 17 different table grape varieties were divided into five classes, A: *Aishenmeigui*, *Zitianwuhe*, *Ruiduwuheyi*, *Sunmmer Black*, *Shaoxing 1 hao* and *black beet*; B: *Ruby Seedless*; C: *Heibaladuo*, *Manicure Finger*, *Sweet Sapphire* and *Hutai 8 hao*; D: *Italy*, *Zhengyanwuhe*, *Zaoheibao* and *Jumeigui*; E: *Meixiangbao* and *Shine-Muscat.* To summarize, the content of total anthocyanins and total phenolics was higher in the category A variety, and the color of fruit skin was black-purple. Thus, this variety was named the purple-black grape. The content of titratable acid in the category B variety was higher, and the maturity was lower, and so it was named the acid grape. The category C variety was named the balanced grape because of its high sensory evaluation and characteristic aroma. The vitamin C and flavonoid content of category D variety was higher. Thus, this variety was named the nutrition grape. The category E variety was named the luzhou-flavor grape because of its rich aroma and high total content. The cluster analysis result is consistent with the content presented by the load diagram of PCA.

## 4. Conclusions

In this study, the basic physical and chemical qualities, nutritional and aroma components, and sensory evaluation data of 17 varieties of table grapes were obtained. The soluble solid content of the new variety *Ruby Seedless* was 21.17%, which was higher than that of other varieties. *Aishenmeigui* and *Sweet Sapphire* had the highest polyphenol and anthocyanin contents. In addition, *Aishenmeigui* was rich in tannins and vitamin C. The flavonoid content in *Heibaladuo* and *Zitianwuhe* was higher than that in other varieties. Aroma contents in *Meixiangbao*, *Ruby Seedless*, and *Shine-Muscat* were higher than in other varieties. *Italia*, *Manicure Finger*, and *Ruby Seedless* had higher levels of C6 compounds. In addition, our results showed that Muscat varieties, including *Zaoheibao*, *Aishenmeigui*, *Jumeigui*, and *Shine-Muscat*, had higher contents of terpenes. Hence, the varieties with better comprehensive quality were *Ruby Seedless*, *Shine-Muscat*, and *Aishenmeigui*. These findings may serve as references for describing the differences in table grape varieties. These germplasm resources can provide valuable genetic resources for breeding fresh grape varieties with better nutritional quality in the future.

## Figures and Tables

**Table 1:** Analysis of the basic physical and chemical quality of different table grapes.

Number	pH	Single Berry Weight (g)	Soluble Solids	Titratable Acid	Longitudinal Diameter	Transverse Diameter
A	4.13 ± 0.02 ^e^	7.07 ± 0.19 ^d^	20.41 ± 0.58 ^b^	7.64 ± 0.07 ^k^	2.59 ± 0.67 ^cd^	1.68 ± 0.03 ^ef^
B	3.04 ± 0.01 ^m^	5.59 ± 0.25 ^e^	16.73 ± 0.24 ^fg^	6.77 ± 0.01 ^a^	2.28 ± 0.11 ^de^	2.04 ± 0.13 ^bc^
C	3.13 ± 0.02 ^l^	7.75 ± 0.24 ^cd^	17.08 ± 0.03 ^f^	8.63 ± 0.02 ^b^	2.65 ± 0.08 ^bd^	2.55 ± 0.09 ^a^
D	4.20 ± 0.02 ^d^	3.90 ± 0.22 ^f^	18.53 ± 0.12 ^d^	8.16 ± 0.03 ^l^	1.96 ± 0.15 ^e^	1.87 ± 0.11 ^ce^
E	3.76 ± 0.01 ^h^	3.76 ± 0.16 ^f^	16.28 ± 0.25 ^ge^	7.99 ± 0.02 ^i^	1.45 ± 0.09 ^i^	1.34 ± 0.08 ^df^
F	3.22 ± 0.03 ^k^	5.76 ± 0.31 ^e^	16.04 ± 0.09 ^e^	8.55 ± 0.13 ^c^	2.37 ± 0.31 ^ce^	2.12 ± 0.31 ^bc^
G	4.02 ± 0.03 ^f^	6.93 ± 0.36 ^d^	18.02 ± 0.12 ^de^	7.77 ± 0.14 ^j^	3.08 ± 0.11 ^ab^	1.73 ± 0.26 ^def^
H	4.45 ± 0.02 ^b^	10.87 ± 1.24 ^a^	19.61 ± 0.06 ^c^	7.92 ± 0.02 ^i^	2.27 ± 0.15 ^de^	2.15 ± 0.12 ^bc^
I	3.34 ± 0.03 ^j^	4.15 ± 0.24 ^f^	16.75 ± 0.12 ^fg^	8.02 ± 0.12 ^b^	1.76 ± 0.11 ^hi^	1.54 ± 0.10 ^ef^
J	3.90 ± 0.01 ^g^	8.82 ± 0.36 ^bc^	15.89 ± 0.21 ^e^	8.41 ± 0.01 ^e^	3.11 ± 0.18 ^a^	2.03 ± 0.11 ^bcd^
K	4.37 ± 0.01 ^b^	7.90 ± 0.07 ^cd^	21.17 ± 0.13 ^a^	8.21 ± 0.02 ^h^	2.42 ± 0.10 ^cd^	1.64 ± 0.12 ^de^
L	4.02 ± 0.05 ^f^	7.33 ± 0.18 ^cd^	20.34 ± 0.29 ^b^	8.36 ± 0.06 ^f^	1.93 ± 0.07 ^e^	1.57 ± 0.09 ^ef^
M	3.85 ± 0.02 ^g^	10.37 ± 0.70 ^a^	17.70 ± 0.19 ^e^	7.37 ± 0.04 ^f^	2.36 ± 0.13 ^ce^	2.13 ± 0.18 ^bc^
N	4.31 ± 0.03 ^a^	8.53 ± 0.24 ^bc^	19.29 ± 0.25 ^c^	8.44 ± 0.02 ^b^	2.52 ± 0.20 ^cd^	2.25 ± 0.19 ^ab^
O	4.10 ± 0.02 ^c^	9.28 ± 0.14 ^b^	20.92 ± 0.11 ^ab^	7.49 ± 0.01 ^d^	2.54 ± 0.08 ^cd^	2.59 ± 0.32 ^a^
P	3.84 ± 0.01 ^g^	8.67 ± 0.32 ^bc^	16.91 ± 0.15 ^f^	8.03 ± 0.08 ^i^	2.63 ± 0.26 ^bd^	2.26 ± 0.13 ^ab^
Q	3.53 ± 0.03 ^i^	7.27 ± 0.19 ^d^	15.71 ± 0.29 ^e^	8.30 ± 0.02 ^g^	2.21 ± 0.06 ^d^	1.76 ± 0.04 ^de^

Note: All values shown are mean ± SD, n = 3. Lower-case letters indicate significant (*p* < 0.05).

**Table 2:** Aroma substance concentrations of 17 different table grape varieties.

Aroma Compounds (μg/L)	Aroma Type	Grape Varieties					
		*Meixiangbao*	*Italy*	*Zaoheibao*	*Manicure Finger*	*Aishenmeigui*	*Zhengyanwuhe*
C_6_ Compounds							
Hexanal		654.29 ± 0.21 ^b^	468.03 ± 1.56 ^d^	547.30 ± 0.06 ^cd^	803.41 ± 2.13 ^a^	237.52 ± 0.04 ^i^	414.01 ± 0.35 ^de^
Hexanol	Fruity	41.56 ± 0.16 ^j^	230.94 ± 9.62 ^a^	77.14 ± 0.37 ^g^	139.65 ± 1.63 ^e^	90.01 ± 3.34 ^f^	52.60 ± 2.60 ^i^
3-Hexanal	Fruity	7.02 ± 0.98 ^c^	0.35 ± 0.01 ^h^	0.32 ± 0.13 ^h^	6.62 ± 0.68 ^d^	6.84 ± 0.03 ^c^	2.13 ± 0.03 ^e^
(E)-2-Hexanal		856.07 ± 7.09 ^e^	1190.67 ± 5.15 ^a^	758.47 ± 18.43 ^fh^	985.13 ± 5.09 ^c^	988.43 ± 10.68 ^c^	970.02 ± 16.58 ^d^
(E)-3-Hexanol		5.51 ± 0.42 ^d^	4.96 ± 0.06 ^f^	9.22 ± 0.98 ^g^	1.96 ± 0.05 ^h^	-	7.89 ± 0.44 ^a^
(E)-2-Hexanol	Grass	221.83 ± 11.73 ^f^	217.75 ± 7.45 ^eg^	105.52 ± 8.61 ^h^	281.29 ± 9.60 ^d^	361.92 ± 7.82 ^a^	98.76 ± 0.38 ^eh^
(Z)-3-Hexanol		0.51 ± 0.02 ^d^	-	0.34 ± 0.11 ^e^	-	-	1.57 ± 0.09 ^a^
Subtotal		1786.79 ± 21.57 ^b^	2112.7 ± 39.66 ^a^	1498.31 ± 18.40 ^e^	2218.06 ± 37.84 ^a^	1684.72 ± 20.35 ^c^	1546.98 ± 44.29 ^cd^
**Alcohols**							
Heptanol	Sweet wine	0.23 ± 0.04 ^f^	-	-	-	-	0.59 ± 0.17 ^d^
Octanol	Nutty	0.76 ± 0.01 c	-	0.94 ± 0.24 ^a^	0.41 ± 0.03 ^e^	-	0.89 ± 0.02 ^a^
Nonanol		-	0.95 ± 0.28 ^a^	-	-	-	-
Benzyl alcohol		0.57 ± 0.03 ^e^	-	0.61 ± 0.05 ^d^	-	-	0.71 ± 0.01 ^c^
Phenylethyl alcohol	Flower	16.01 ± 1.41 ^c^	6.22 ± 0.06 ^g^	24.32 ± 3.62 ^a^	3.08 ± 0.77 ^k^	4.28 ± 0.08 ^i^	7.12 ± 1.23 ^e^
2-Heptanol		-	-	-	-	-	1.28 ± 0.06 ^b^
1-Octen-3-ol		0.44 ± 0.02 ^e^	0.99 ± 0.15 ^a^	0.39 ± 0.06 ^e^	-	-	0.73 ± 0.02 ^cd^
2-Ethyl hexanol	Flower	2.31 ± 0.01 ^de^	3.11 ± 0.62 ^d^	2.89 ± 0.38 ^e^	2.72 ± 0.85 ^e^	5.08 ± 0.04 ^a^	3.01 ± 0.71 ^d^
Subtotal		20.72 ± 3.28 ^c^	11.38 ± 0.49 ^f^	29.20 ± 0.61 ^b^	6.38 ± 1.07 ^k^	9.36 ± 2.23 ^h^	14.43 ± 1.25 ^e^
**Esters**							
Etnyl acetate	Vegetable oil	3056.59 ± 22.14 ^d^	2716.64 ± 50.08 ^e^	347.97 ± 6.72 ^jk^	986.28 ± 22.13 ^k^	473.34 ± 17.09 ^l^	1031.81 ± 35.61 ^f^
Ethyl propionate	Pineapple	10.01 ± 0.29 ^b^	-	-	3.91 ± 0.03 ^g^	-	0.85 ± 0.01 ^i^
Propyl acetate		0.36 ± 0.07 ^e^	-	0.57 ± 0.03 ^bc^	-	-	0.45 ± 0.06 ^c^
Ethyl butyrate	Apple; Banana	209.94 ± 10.30 ^b^	210.73 ± 8.46 ^bc^	96.36 ± 5.14 ^l^	163.41 ± 7.54 ^d^	142.21 ± 10.71 ^ef^	30.53 ± 4.08 ^j^
Butyl acetate	Fruity	0.84 ± 0.06 ^a^	0.49 ± 0.09 ^c^	-	0.39 ± 0.01 ^d^	0.52 ± 0.12 ^c^	0.63 ± 0.23 ^b^
Ethyl pentanoate		3.08 ± 0.71 ^a^	-	1.54 ± 0.02 ^c^	2.91 ± 0.15 ^ab^	-	-
Methyl hexanoate		1.24 ± 0.06 ^d^	-	4.01 ± 1.21 ^a^	-	-	0.82 ± 0.01 e
Ethyl hexanoate		23.14 ± 2.37 ^ab^	-	-	22.34 ± 3.61 ^b^	-	-
Hexyl acetate		-	3.23 ± 0.06 ^b^	1.82 ± 0.02 ^d^	-	-	3.91 ± 0.62 ^a^
Ethyl heptanoate	Rose	9.04 ± 2.31 ^a^	1.52 ± 0.58 ^cde^	-	2.06 ± 0.49 ^b^	0.54 ± 0.01 ^g^	0.74 ± 0.11 ^g^
Ethyl octanoate		2.21 ± 0.05 ^c^	-	-	-	-	-
Ethyl isobutyrate	Strawberry	-	7.23 ± 0.14 ^c^	1.39 ± 0.25 ^g^	4.45 ± 0.81 ^e^	-	0.88 ± 0.04
Benzoic acid ethyl ester		-	3.98 ± 0.28 ^d^	0.24 ± 0.01 ^j^	3.92 ± 0.76 ^d^	3.06 ± 0.39 ^e^	-
Methyl salicylate		-	9.13 ± 1.02 ^b^	0.17 ± 0.02 ^i^	-	-	-
Methyl anthranilate		4.37 ± 0.25 ^bc^	5.45 ± 0.31 ^b^	0.23 ± 0.02 ^j^	0.53 ± 0.06 ^i^	-	0.61 ± 0.21 ^hi^
Ethyl-2-methylbutanoate	Fruity	3.05 ± 0.17 ^d^	-	-	-	-	-
Ethyl-3-methylbutanoate		0.31 ± 0.01 ^j^	-	1.79 ± 0.36 ^g^	6.73 ± 0.28 ^b^	-	0.13 ± 0.01 ^j^
(Z)-2-Butenoic acid, ethyl ester		8.06 ± 1.32 ^b^	9.19 ± 1.46 ^ab^	-	2.78 ± 0.57 ^f^	-	-
2-Hexenoic acid, ethyl ester		3.36 ± 0.58 ^c^	-	-	-	1.34 ± 0.01 ^e^	0.92 ± 0.15 ^ef^
Ethyl-3-hydroxybutyrate	Wine	1.75 ± 0.07 ^d^	3.26 ± 0.02 ^c^	0.83 ± 0.11 ^f^	-	-	0.95 ± 0.03 ^f^
Subtotal		3336.24 ± 56.71 ^b^	2970.93 ± 34.28 ^cd^	456.94 ± 7.06 ^n^	1199.71 ± 24.29 ^hij^	621.01 ± 12.51 ^km^	1073.23 ± 23.64 ^j^
**Acids**		-	-	-	-	-	-
Hexanoic acid		-	-	-	1.31 ± 0.03 ^c^	-	1.59 ± 0.21 ^b^
Nonanoic acid		-	0.55 ± 0.01 ^c^	-	0.75 ± 0.12 ^b^	-	0.53 ± 0.01 ^c^
2-Hexenoic		1.24 ± 0.03 ^d^	-	-	-	-	1.36 ± 0.05 ^d^
Subtotal		1.24 ± 0.03 ^d^	0.55 ± 0.01 ^f^	0	2.06 ± 0.21 ^c^	0	3.48 ± 0.34 ^a^
**Aldehydes**							
Pentanal		-	5.21 ± 0.35 ^a^	-	3.98 ± 0.61 ^c^	1.05 ± 0.02 ^g^	-
Heptanal	Herb	0.44 ± 0.09 ^k^	13.07 ± 1.22 ^bc^	2.40 ± 0.09 ^h^	1.53 ± 0.25 ^i^	-	0.67 ± 0.01 ^jk^
Octanal		-	-	4.66 ± 1.07 ^c^	-	-	-
Nonanal		1.09 ± 0.51 ^g^	3.91 ± 0.37 ^cd^	5.80 ± 0.62 ^a^	6.46 ± 0.47 ^a^	-	0.57 ± 0.03 ^h^
Decanal	Citrus peel	-	-	-	-	2.51 ± 0.31 ^e^	3.15 ± 1.01 ^d^
Benzaldehyde		8.93 ± 1.42 ^a^	0.36 ± 0.02 ^h^	8.02 ± 0.09 ^ab^	-	-	-
Phenylacetaldehyde	Hyacinth	4.65 ± 0.26 ^f^	-	9.24 ± 1.51 ^c^	4.07 ± 0.02 ^fg^	1.59 ± 0.34 ^i^	7.94 ± 1.63 ^d^
(Z)-2-hepental		-	-	-	-	8.51 ± 2.30 ^c^	-
(E)-2-nonenal		-	9.18 ± 0.76 ^a^	-	7.85 ± 0.05 ^bc^	1.65 ± 0.81 ^j^	6.29 ± 1.45 ^cd^
Subtotal		15.11 ± 1.68 ^j^	31.66 ± 0.91 ^cd^	30.12 ± 1.60 ^def^	24.31 ± 0.09 ^e^	15.31 ± 3.24 ^ij^	18.62 ± 3.06 ^i^
**Terpenes**		-	-	-	-	-	-
α-Pinene	Resin	-	8.02 ± 0.24 ^i^	-	80.23 ± 11.08 ^bc^	0.65 ± 0.02 ^l^	44.81 ± 5.67 ^g^
β-Pinene	Resin	5.14 ± 0.57 ^ijkl^	13.26 ± 1.08 ^ef^	96.36 ± 12.51 ^a^	-	5.72 ± 2.08 ^ijk^	19.28 ± 3.11 ^d^
Eucalyptol		6.27 ± 0.03 ^jkl^	8.76 ± 1.12 ^jk^	-	55.35 ± 2.45 ^ab^	25.18 ± 3.51 ^def^	27.20 ± 0.59 ^de^
Nerol ooxide		-	2.93 ± 0.06 ^l^	79.69 ± 4.71 ^bc^	76.57 ± 6.83 ^c^	9.14 ± 0.04 ^k^	14.86 ± 0.24 ^i^
Linalool		38.09 ± 5.62 ^ij^	27.81 ± 3.02 ^k^	155.89 ± 12.97 ^a^	94.06 ± 10.55 ^fg^	139.53 ± 8.65 ^bc^	-
Neral	Grass	-	4.01 ± 0.06 ^n^	58.34 ± 4.51 ^d^	75.80 ± 6.59 ^a^	11.78 ± 0.56 ^k^	16.27 ± 0.34 ^j^
Geranial cis-Linalool oxide	Rose	4.61 ± 0.51 ^l^	17.74 ± 0.94 ^i^	44.01 ± 5.77 ^e^	61.18 ± 5.90 ^c^	10.86 ± 2.81 ^j^	49.98 ± 0.46 ^e^
Citral Linalool oxide		7.93 ± 1.32 ^jk^	14.93 ± 2.08 ^ijk^	78.45 ± 4.89 ^de^	32.25 ± 6.34 ^f^	6.72 ± 1.06 ^jkl^	5.12 ± 0.91 ^l^
Citronellol		-	7.35 ± 1.22 ^m^	562.71 ± 33.40 ^c^	380.55 ± 19.67 ^e^	19.25 ± 0.05 ^k^	4.29 ± 1.07 ^klm^
Geranic acid	Vegetables	213.16 ± 12.64 ^b^	18.44 ± 3.19 ^l^	56.92 ± 3.51 ^j^	72.53 ± 7.12 ^i^	262.30 ± 22.61 ^a^	115.94 ± 8.59 ^g^
α-Phellandrene		14.55 ± 3.21 ^f^	9.83 ± 1.75 ^h^	-	-	7.54 ± 0.07 ^j^	29.23 ± 2.34 ^d^
α-Terpinene		-	-	1.21 ± 0.05 ^l^	66.39 ± 4.55 ^b^	34.87 ± 4.35 ^e^	20.40 ± 3.63 ^h^
α-Terpineol	Flowers	-	14.62 ± 0.24 ^j^	-	88.82 ± 5.06 ^c^	-	41.16 ± 5.29 ^g^
β-Myrcene	Grass	3.49 ± 0.02 ^l^	9.88 ± 0.33 ^i^	0.51 ± 0.01 ^mn^	37.36 ± 2.48 ^e^	3.34 ± 0.52 ^l^	49.58 ± 10.51 ^cd^
β-Ocimene		-	-	20.08 ± 3.43 ^bcd^	21.53 ± 3.49 ^bc^	24.50 ± 3.42 ^b^	-
(Z)-Limonene		-	4.82 ± 2.51 ^hijk^	99.34 ± 11.59 ^a^	-	5.79 ± 0.80 ^hi^	57.87 ± 12.69 ^de^
γ-Terpinene	Lemon	5.61 ± 1.32 ^jk^	4.01 ± 0.02 ^kl^	46.78 ± 4.56 ^d^	57.54 ± 7.51 ^a^	34.03 ± 5.62 ^cd^	8.81 ± 0.03 ^j^
4-Terpinene		-	-	83.55 ± 7.03 ^a^	-	11.63 ± 1.13 ^j^	-
o-Cymene		15.13 ± 2.62 ^bc^	7.09 ± 0.34 ^g^	-	-	13.26 ± 0.21 ^cde^	49.34 ± 0.06 ^a^
m-Cymene		8.12 ± 0.04 ^i^	5.85 ± 0.02 ^j^	71.43 ± 3.25 ^b^	1.68 ± 0.03 ^l^	6.91 ± 0.44 ^i^	37.73 ± 3.18 ^e^
Terpinolene	Nutty	-	15.06 ± 1.71 ^fgh^	59.05 ± 0.47 ^bc^	1.02 ± 0.01 ^hi^	24.96 ± 2.61 ^f^	69.16 ± 5.51 ^b^
Eugenol	Lilac	-	11.68 ± 2.09 ^g^	91.95 ± 2.36 ^a^	-	-	31.80 ± 0.72 ^d^
Hotrienol		3.68 ± 0.47 ^hi^	-	34.51 ± 3.21 ^bc^	1.43 ± 0.07 ^h^	36.66 ± 3.49 ^b^	7.56 ± 0.05 ^g^
Myrtenol	Mint	-	14.37 ± 1.53 ^gh^	-	36.67 ± 1.22 ^c^	28.66 ± 0.51 ^cde^	-
Isogeraniol		9.78 ± 2.05 ^i^	-	15.60 ± 1.46 ^hi^	56.32 ± 6.05 ^cd^	8.94 ± 0.12 ^jk^	43.79 ± 2.37 ^efg^
E-Nerolidol		-	13.41 ± 1.51 ^gh^	35.82 ± 2.34 ^de^	-	6.73 ± 0.61 ^j^	12.16 ± 0.72 ^ghi^
cis-β-Ocimene		0.42 ± 0.02 ^k^	18.62 ± 2.34 ^h^	74.76 ± 6.21 ^b^	82.14 ± 4.59 ^a^	15.42 ± 0.32 ^h^	53.59 ± 0.81 ^d^
trans-β-Ocimene	Citrus	-	7.83 ± 0.21 ^j^	28.14 ± 3.76 ^g^	69.76 ± 5.32 ^d^	20.06 ± 2.27 ^h^	24.56 ± 2.65 ^g^
cis-Rose oxide	Roses	41.29 ± 3.54 ^b^	3.55 ± 0.34 ^e^	-	-	31.51 ± 4.15 ^c^	-
trans-Rose oxide	Litchi	12.06 ± 2.11 ^l^	6.18 ± 3.15 ^m^	67.06 ± 7.54 ^e^	96.35 ± 12.31 ^c^	13.40 ± 0.04 ^l^	31.36 ± 2.16 ^i^
Subtotal		389.87 ± 18.54 ^mn^	270.45 ± 21.01 ^p^	1864.32 ± 32.65 ^de^	995.42 ± 21.83 ^hi^	819.69 ± 22.51 ^k^	871.79 ± 33.41 ^ijk^
**C13-Norisoprenoids**		-	-	-	-	-	-
β-Damascenone	Honey	0.24 ± 0.02 ^f^	0.64 ± 0.06 ^ef^	0.96 ± 0.03 ^cd^	-	1.86 ± 0.21 ^a^	-
β-Ionone	Violets	-	0.51 ± 0.03 ^cd^	0.34 ± 0.02 ^e^	0.92 ± 0.06 ^b^	1.43 ± 0.02 ^ab^	0.44 ± 0.03 ^de^
Geranylacetone	Flowers	-	0.81 ± 0.05 ^c^	0.95 ± 0.13 ^b^	0.87 ± 0.06 ^c^	1.14 ± 0.01 ^b^	-
Subtotal		0.45 ± 0.05 ^i^	1.96 ± 0.03 ^e^	2.25 ± 0.15 ^de^	1.79 ± 0.02 ^e^	4.43 ± 0.31 ^a^	1.18 ± 0.12 ^efg^
TOTAL		7548.73 ± 46.51 ^b^	5397.12 ± 123.17 ^de^	3878.89 ± 84.06 ^ij^	4995.88 ± 79.31 ^efg^	3150.27 ± 36.12 ^j^	3525.05 ± 84.49 ^ij^
**Aroma Compounds (μg/L)**	**Aroma Type**	**Grape Varieties**					
		***Heibaladuo***	***Ruby Seedless***	***Ruiduwuheyi***	***Sweet Sapphire***	***Shaoxing 1 Hao***	
**C_6_ Compounds**							
Hexanal		307.41 ± 6.71 ^gh^	462.23 ± 1.08 ^cd^	413.55 ± 0.54 ^e^	655.13 ± 3.65 ^b^	374.01 ± 2.80 ^f^	
Hexanol	Fruity	156.82 ± 0.52 ^d^	157.49 ± 2.49 ^d^	52.14 ± 5.74 ^i^	42.40 ± 2.23 ^j^	62.93 ± 5.74 ^h^	
3-Hexanal	Fruity	9.04 ± 0.16 ^a^	9.71 ± 1.17 ^a^	1.67 ± 0.05 ^f^	7.86 ± 0.67 ^b^	1.59 ± 0.02 ^f^	
(E)-2-Hexanal		1124.60 ± 13.48 ^a^	1025.27 ± 8.44 ^b^	969.56 ± 9.51 ^d^	856.91 ± 13.14 ^e^	836.57 ± 9.30 ^ef^	
(E)-3-Hexanol		-	-	7.43 ± 1.15 ^b^	6.35 ± 0.03 ^c^	5.46 ± 0.65 ^e^	
(E)-2-Hexanol	Grass	329.68 ± 8.43 ^c^	370.35 ± 6.12 ^a^	98.30 ± 7.54 ^h^	222.67 ± 8.62 ^g^	356.45 ± 12.73 ^b^	
(Z)-3-Hexanol		-	-	1.11 ± 0.03 ^c^	1.35 ± 0.21 ^b^	-	
Subtotal		1927.55 ± 28.12 ^bc^	2085.06 ± 15.77 ^b^	1546.52 ± 10.12 ^ef^	1787.63 ± 12.06 ^d^	1408.27 ± 17.32 ^f^	
**Alcohols**							
Heptanol	Sweet wine	-	0.70 ± 0.01 ^b^	-	1.07 ± 0.23 ^a^	0.63 ± 0.11 ^cd^	
Octanol	Nutty	-	0.67 ± 0.04 ^cd^	0.40 ± 0.01 ^e^	0.31 ± 0.02 ^efg^	0.82 ± 0.03 ^b^	
Nonanol		0.52 ± 0.02 ^bc^	1.19 ± 0.33 ^a^	0.46 ± 0.05 ^bc^	-	-	
Benzyl alcohol		-	-	0.25 ± 0.04 ^e^	1.41 ± 0.22 ^a^	-	
Phenylethyl alcohol	Flower	8.49 ± 0.12 ^d^	9.16 ± 1.21 ^d^	6.63 ± 0.65 ^f^	16.85 ± 2.09 ^b^	10.08 ± 0.43 ^d^	
2-Heptanol		0.17 ± 0.01 ^fg^	-	0.82 ± 0.04 ^a^	-	-	
1-Octen-3-ol		0.59 ± 0.02 ^efg^	1.26 ± 0.02 ^a^	0.27 ± 0.01 ^i^	1.28 ± 0.14 ^a^	1.14 ± 0.02 ^b^	
2-Ethyl hexanol	Flower	2.04 ± 0.35 ^cd^	2.71 ± 0.07 ^bc^	2.65 ± 0.21 ^c^	3.55 ± 0.32 ^b^	0.93 ± 0.03 ^h^	
Subtotal		11.84 ± 1.26 ^j^	12.51 ± 1.27 ^g^	13.97 ± 2.35 ^f^	21.56 ± 3.87 ^abc^	12.07 ± 5.76 ^gh^	
**Esters**							
Etnyl acetate	Vegetable oil	2006.28 ± 19.51 ^f^	5086.95 ± 26.87 ^a^	1031.35 ± 10.71 ^g^	3057.43 ± 23.10 ^c^	2517.36 ± 15.46 ^de^	
Ethyl propionate	Pineapple	-	-	0.31 ± 0.02 ^fg^	2.18 ± 0.65 ^a^	0.49 ± 0.04 ^f^	
Propyl acetate		-	0.36 ± 0.06 ^d^	-	-	-	
Ethyl butyrate	Apple; banana	136.54 ± 2.98 ^cd^	141.21 ± 8.75 ^c^	30.07 ± 4.81 ^jk^	210.77 ± 15.06 ^a^	37.54 ± 0.27 ^ij^	
Butyl acetate	Fruity	0.77 ± 0.02 ^e^	1.44 ± 0.16 ^c^	-	1.68 ± 0.15 ^b^	-	
Ethyl pentanoate		-	-	-	3.92 ± 0.07 ^c^	-	
Methyl hexanoate		-	-	0.36 ± 0.05 ^e^	2.08 ± 0.62 ^b^	-	
Ethyl hexanoate		0.17 ± 0.02 ^k^	0.74 ± 0.23 ^gh^	-	23.49 ± 3.12 ^a^	0.82 ± 0.02 ^g^	
Hexyl acetate		0.52 ± 0.04 ^e^	1.19 ± 0.51 ^c^	3.45 ± 0.62 ^a^	-	-	
Ethyl heptanoate	Rose	-	0.67 ± 0.02 ^f^	0.28 ± 0.03 ^f^	9.84 ± 1.34 ^b^	-	
Ethyl octanoate		0.37 ± 0.02 ^gh^	1.04 ± 0.08 ^g^	-	3.51 ± 0.62 ^e^	12.13 ± 1.65 ^a^	
Ethyl isobutyrate	Strawberries	0.28 ± 0.03 ^j^	0.95 ± 0.05 ^i^	0.42 ± 0.01 ^ij^	-	8.77 ± 0.53 ^cd^	
Benzoic acid ethyl ester		0.84 ± 0.16 ^c^	1.51 ± 0.19 ^a^	-	-	-	
Methyl salicylate		-	-	-	-	20.35 ± 2.26 ^c^	
Methyl anthranilate		-	0.75 ± 0.01 ^i^	0.15 ± 0.01 ^k^	5.21 ± 0.12 ^fg^	7.13 ± 1.04 ^d^	
Ethyl 2-methylbutanoate	Fruity	2.58 ± 0.51 ^ab^	2.99 ± 0.16 ^a^	-	-	-	
Ethyl 3-methylbutanoate		0.57 ± 0.05 ^d^	0.94 ± 0.04 ^c^	-	-	-	
(Z)-2-Butenoic acid, ethyl ester		-	-	-	8.09 ± 0.07 ^c^	9.49 ± 1.56 ^b^	
2-Hexenoic acid, ethyl ester		0.48 ± 0.04 ^d^	0.95 ± 0.06 ^b^	-	-	-	
Ethyl-3-hydroxybutyrate	Wine	0.55 ± 0.05 ^g^	1.22 ± 0.13 ^e^	0.49 ± 0.02 ^gh^	2.59 ± 0.64 ^de^	6.44 ± 1.27 ^b^	
Subtotal		2131.25 ± 76.42 ^h^	5731.92 ± 45.89 ^b^	2970.93 ± 62.71 ^fg^	3337.08 ± 15.02 ^f^	784.61 ± 20.82 ^jk^	
**Acids**							
Hexanoic acid		2.39 ± 0.05 ^e^	3.06 ± 0.24 ^d^	1.13 ± 0.45 ^g^	-	0.72 ± 0.02 ^j^	
Nonanoic acid		0.81 ± 0.02 ^g^	1.48 ± 0.12 ^f^	-	-	-	
2-Hexenoic		-	-	0.91 ± 0.05 ^d^	2.08 ± 0.56 ^c^	0.43 ± 0.02 ^d^	
Subtotal		3.20 ± 0.51 ^d^	3.87 ± 0.64 ^bc^	3.04 ± 0.92 ^de^	2.08 ± 0.56 ^f^	3.62 ± 1.54 ^c^	
**Aldehydes**							
Pentanal		-	-	-	-	6.76 ± 1.03 ^b^	
Heptanal	Herb	4.87 ± 0.76 ^b^	5.54 ± 1.04 ^a^	0.21 ± 0.02 ^f^	1.28 ± 0.06 ^e^	0.51 ± 0.04 ^f^	
Octanal		0.66 ± 0.02 ^d^	1.33 ± 0.05 ^b^	-	-	-	
Nonanal		-	-	-	1.93 ± 0.62 ^f^	4.65 ± 0.61 ^d^	
Decanal	Citrus Peel	-	-	2.69 ± 0.74 ^c^	-	-	
Benzaldehyde		3.45 ± 0.07 ^h^	4.12 ± 0.60 ^g^	-	9.77 ± 1.25 ^c^	5.96 ± 0.03 ^f^	
Phenylacetaldehyde	Hyacinth	1.05 ± 0.02 ^ij^	1.72 ± 0.43 ^i^	7.48 ± 0.22 ^b^	5.49 ± 1.01 ^d^	4.19 ± 0.05 ^ef^	
(Z)-2-hepental		0.21 ± 0.01 ^d^	0.88 ± 0.01 ^c^	-	-	-	
(E)-2-nonenal		5.65 ± 0.92 ^b^	6.32 ± 0.33 ^a^	5.83 ± 0.07 ^b^	-	4.68 ± 0.16 ^e^	
Subtotal		15.89 ± 1.43 ^f^	16.56 ± 2.01 ^e^	18.16 ± 2.17 ^d^	15.95 ± 3.50 ^f^	27.31 ± 4.51 ^b^	
**Terpenes**		-				-	
α-Pinene	Resin	11.07 ± 0.47 ^efg^	11.74 ± 1.32 ^ef^	44.35 ± 5.64 ^a^	-	12.99 ± 0.85 ^e^	
β-Pinene	Resin	17.08 ± 1.20 ^ab^	18.75 ± 2.16 ^a^	8.82 ± 0.59 ^de^	5.98 ± 0.04 ^g^	7.10 ± 1.03 ^ef^	
Eucalyptol		15.38 ± 0.54 ^cd^	16.05 ± 0.22 ^c^	36.74 ± 2.17 ^b^	7.11 ± 0.42 ^hi^	5.42 ± 0.06 ^i^	
Nerol ooxide		14.39 ± 0.32 ^ab^	15.06 ± 0.54 ^a^	13.04 ± 0.02 ^b^	-	-	
Linalool		10.04 ± 1.58 ^e^	10.71 ± 0.02 ^e^	-	38.93 ± 2.40 ^a^	-	
Neral	Grass	17.51 ± 1.22 ^a^	-	15.81 ± 1.55 ^c^	-	-	
Geranial cis-Linalool oxide	Rose	1.44 ± 0.04 ^j^	2.11 ± 0.08 ^j^	49.52 ± 3.40 ^a^	5.45 ± 0.08 ^g^	7.62 ± 0.37 ^f^	
Citral Linalool oxide		12.50 ± 1.33 ^e^	15.17 ± 2.21 ^d^	4.66 ± 0.05 ^i^	8.77 ± 0.21 ^g^	25.86 ± 2.20 ^b^	
Citronellol		415.77 ± 33.41 ^cd^	616.44 ± 15.84 ^a^	-	-	84.58 ± 8.71 ^g^	
Geranic acid	Vegetables	10.58 ± 0.91 ^h^	11.25 ± 0.49 ^h^	105.48 ± 7.34 ^d^	214.00 ± 3.01 ^a^	18.13 ± 0.06 ^f^	
α-Phellandrene		3.19 ± 1.43 ^k^	6.86 ± 0.72 ^hij^	28.77 ± 2.31 ^c^	15.39 ± 0.31 ^ef^	8.84 ± 0.95 ^h^	
α-Terpinene		4.40 ± 0.02 ^e^	5.65 ± 1.01 ^d^	19.94 ± 0.55 ^a^	-	3.06 ± 0.30 ^e^	
α-Terpineol	Flower	3.73 ± 0.01 ^g^	4.24 ± 0.05 ^g^	40.71 ± 1.62 ^b^	-	-	
β-Myrcene	Grass	10.26 ± 0.03 ^c^	12.93 ± 1.43 ^b^	-	4.33 ± 0.65 ^f^	2.94 ± 0.02 ^gh^	
β-Ocimene		-	8.31 ± 0.10 ^e^	-	-	19.15 ± 1.76 ^a^	
(Z)-Limonene		-	5.41 ± 0.16 ^fg^	-	-	14.20 ± 3.04 ^c^	
γ-Terpinene	Lemon	1.41 ± 0.03 ^gh^	2.08 ± 0.06 ^g^	8.35 ± 0.06 ^bc^	6.45 ± 1.03 ^d^	-	
4-Terpinene		-	-	-	-	-	
o-Cymene		4.83 ± 0.54 ^g^	8.50 ± 1.22 ^f^	48.88 ± 3.02 ^a^	15.97 ± 0.06 ^d^	10.42 ± 1.15 ^e^	
m-Cymene		-	1.71 ± 0.53 ^gh^	37.27 ± 2.19 ^b^	8.96 ± 0.29 ^ef^	4.80 ± 0.04 ^g^	
Terpinolene	Nutty	-	-	-	-	22.23 ± 2.07 ^b^	
Eugenol	Lilac	8.07 ± 0.09 ^c^	8.74 ± 0.35 ^b^	0.34 ± 0.06 ^j^	-	-	
Hotrienol		13.45 ± 1.27 ^cd^	17.12 ± 0.04 ^bc^	7.10 ± 0.05 ^g^	4.52 ± 0.31 ^h^	16.95 ± 1.41 ^c^	
Myrtenol	Mint	-	0.67 ± 0.01 ^h^	-	-	7.24 ± 0.05 ^a^	
Isogeraniol		-	18.88 ± 1.34 ^g^	43.33 ± 2.15 ^d^	10.62 ± 1.43 ^hi^	-	
E-Nerolidol		12.13 ± 1.72 ^d^	12.80 ± 0.56 ^d^	11.67 ± 0.94 ^de^	-	16.22 ± 0.65 ^c^	
cis-β-Ocimene		-	-	53.13 ± 4.62 ^b^	1.26 ± 0.03 ^f^	-	
trans-β-Ocimene	Citrus	-	-	24.10 ± 3.47 ^d^	-	10.50 ± 2.07 ^g^	
cis-Rose oxide	Rose	17.15 ± 1.89 ^g^	17.82 ± 1.23 ^g^	-	42.13 ± 3.14 ^c^	20.97 ± 0.51 ^f^	
trans-Rose oxide	Litchi	18.91 ± 2.52 ^g^	29.58 ± 4.61 ^e^	30.91 ± 3.62 ^e^	13.44 ^i^	-	
Subtotal		679.50 ± 22.64 ^ef^	800.17 ± 20.05 ^d^	571.33 ± 44.72 ^g^	390.71 ± 15.23 ^ij^	300.45 ± 8.32 ^k^	
**C13-Norisoprenoids**		-				-	
β-Damascenone	Honey	-	2.24 ± 0.24 ^a^	-	1.08 ± 0.03 ^d^	0.72 ± 0.07 ^de^	
β-Ionone	Violets	1.65 ± 0.05 ^d^	2.32 ± 0.07 ^c^	-	1.05 ± 0.06 ^f^	-	
Geranylacetone	Flower	1.41 ± 0.03 ^c^	2.08 ± 0.01 ^b^	-	-	-	
Subtotal		4.63 ± 0.11 ^bc^	6.64 ± 1.05 ^a^	0	1.29 ± 0.10 ^f^	0.72 ± 0.07 ^fg^	
TOTAL		5839.03 ± 54.91 ^de^	8766.91 ± 122.41 ^a^	3524.59 ± 22.51 ^ijk^	5549.57 ± 72.82 ^e^	2836.64 ± 22.26 ^k^	
**Aroma Compounds (μg/L)**	**Aroma Type**	**Grape Varieties**					
		**Jumeigui**	**Shine-Muscat**	**Hutai 8 Hao**	**Heisetiancai**	**Zitianwuhe**	**Sunmmer Black**
**C_6_ Compounds**							
Hexanal		237.52 ± 3.17 ^i^	307.41 ± 7.48 ^gh^	276.51 ± 1.05 ^hi^	354.71 ± 12.36 ^f^	286.51 ± 0.12 ^h^	167.15 ± 0.91 ^jk^
Hexanol	Fruity	90.01 ± 0.16 ^f^	156.82 ± 4.20 ^d^	201.28 ± 7.91 ^b^	72.71 ± 0.90 ^g^	190.28 ± 4.63 ^c^	159.82 ± 3.23 ^d^
3-Hexanal	Fruity	6.84 ± 0.09 ^cd^	9.04 ± 0.96 ^a^	-	1.19 ± 0.13 ^g^	-	0.12 ± 0.01 ^j^
(E)-2-Hexanal		988.43 ± 9.23 ^c^	1124.60 ± 7.57 ^a^	863.19 ± 5.00 ^e^	726.65 ± 3.16 ^i^	813.19 ± 8.91 ^fg^	703.91 ± 16.32 ^ijk^
(E)-3-Hexanol		-	-	0.25 ± 0.01 ^h^	5.06 ± 0.24 ^d^	-	-
(E)-2-Hexanol	Grass	361.92 ± 10.51 ^a^	329.68 ± 6.33 ^c^	83.81 ± 0.22 ^i^	357.45 ± 15.62 ^b^	63.85 ± 4.72 ^j^	243.60 ± 3.52 ^e^
(Z)-3-Hexanol		-	-	-	-	-	-
Subtotal		1684.72 ± 44.81 ^d^	1927.55 ± 36.02 ^b^	1425.16 ± 22.75 ^e^	1498.17 ± 46.75 ^e^	1354.25 ± 29.03 ^f^	1252.31 ± 30.40 ^g^
**Alcohols**							
Heptanol	Sweet wine	-	-	0.37 ± 0.01 ^e^	0.63 ± 0.03 ^bcd^	0.71 ± 0.04 ^b^	-
Octanol	Nutty	-	-	0.65 ± 0.02 ^d^	0.82 ± 0.11 ^b^	0.64 ± 0.02 ^d^	1.25 ± 0.02 ^b^
Nonanol		-	0.52 ± 0.12 ^c^	-	-	0.42 ± 0.04 ^d^	-
Benzyl alcohol		-	-	-	-	-	-
Phenylethyl alcohol	Flower	4.08 ± 0.55 ^gh^	8.49 ± 0.34 ^b^	9.19 ± 0.62 ^a^	8.01 ± 0.55 ^bc^	6.53 ± 0.74 ^e^	4.95 ± 0.61 ^g^
2-Heptanol		-	0.17 ± 0.01 ^e^	-	-	-	-
1-Octen-3-ol		-	0.59 ± 0.02 ^c^	-	0.81 ± 0.03 ^a^	0.62 ± 0.06 ^c^	-
2-Ethyl hexanol	Flower	5.08 ± 0.07 ^b^	1.84 ± 0.01 ^f^	2.07 ± 0.03 ^f^	3.93 ± 0.05 ^e^	1.47 ± 0.12 ^g^	6.20 ± 0.35 ^a^
Subtotal		9.54 ± 1.21 ^g^	11.84 ± 1.25 ^de^	12.40 ± 2.31 ^d^	14.40 ± 0.59 ^b^	10.45 ± 0.26 ^f^	11.42 ± 0.49 ^e^
**Esters**							
Etnyl acetate	Vegetable oil	473.34 ± 15.66 ^l^	686.28 ± 22.40 ^i^	540.97 ± 15.39 ^k^	470.53 ± 10.54 ^kl^	541.28 ± 20.13 ^k^	490.91 ± 13.47 ^jk^
Ethyl propionate	Pineapple	-	-	2.18 ± 0.03 ^bc^	-	2.49 ± 0.02 ^b^	1.36 ± 0.03 ^f^
Ethyl butyrate	Banana	142.21 ± 2.46 ^c^	140.54 ± 3.08 ^c^	157.27 ± 1.67 ^b^	57.59 ± 2.02 ^j^	137.58 ± 1.80 ^cd^	105.42 ± 2.31 ^g^
Butyl acetate	Fruity	0.52 ± 0.02 ^i^	0.77 ± 0.03 ^g^	-	0.91 ± 0.03 ^d^	0.98 ± 0.02 ^c^	1.13 ± 0.01 ^c^
Ethyl pentanoate		-	-	0.67 ± 0.01 ^d^	-	-	0.82 ± 0.02 ^c^
Methyl hexanoate		-	-	6.05 ± 0.36 ^ab^	-	6.46 ± 0.41 ^a^	3.41 ± 0.13 ^e^
Ethyl hexanoate		-	0.17 ± 0.01 ^g^	15.06 ± 0.02 ^b^	-	11.37 ± 0.75 ^d^	-
Hexyl acetate		-	0.52 ± 0.02 ^h^	-	-	1.03 ± 0.03 ^f^	0.74 ± 0.11 ^g^
Ethyl heptanoate	Rose	0.54 ± 0.01 ^i^	-	5.81 ± 0.65 ^b^	-	6.12 ± 0.83 ^b^	-
Ethyl octanoate		-	0.37 ± 0.02 ^k^	-	12.13 ± 0.19 ^a^	-	-
Ethyl isobutyrate	Strawberries	-	-	13.09 ± 1.06 ^b^	8.77 ± 0.07 ^f^	8.40 ± 0.31 ^f^	10.26 ± 1.09 ^d^
Benzoic acid ethyl ester		3.06 ± 0.13 ^d^	0.84 ± 0.02 ^h^	-	-	-	-
Methyl salicylate		-	5.06 ± 0.70 ^e^	-	10.02 ± 0.40 ^b^	-	-
Methyl anthranilate		-	-	-	6.91 ± 0.05 ^f^	-	12.51 ± 0.23 ^c^
Ethyl 2-methylbutanoate	Fruity	-	-	10.58 ± 1.10 ^a^	-	-	6.35 ± 0.63 ^f^
Ethyl 3-methylbutanoate	Fruity	-	0.57 ± 0.03 ^j^	13.42 ± 0.61 ^c^	-	10.03 ± 0.26 ^de^	-
(Z)-2-Butenoic acid, ethyl ester		-	4.21 ± 0.05 ^g^	-	9.16 ± 0.06 ^c^	-	-
2-Hexenoic acid, ethyl ester		1.34 ± 0.04 ^i^	0.28 ± 0.02 ^k^	12.81 ± 1.42 ^ab^	-	13.12 ± 0.03 ^a^	-
Ethyl-3-hydroxybutyrate	Wine	-	0.55 ± 0.01 ^i^	-	8.31 ± 1.05 ^c^	-	5.77 ± 0.41 ^e^
Subtotal		561.01 ± 12.10 ^hi^	773.25 ± 22.43 ^d^	698.01 ± 10.65 ^ef^	584.33 ± 8.70 ^h^	724.32 ± 15.91 ^de^	534.61 ± 7.49 ^ij^
**Acids**							
Hexanoic acid		-	2.39 ± 0.03 ^a^	-	0.57 ± 0.01 ^f^	-	-
Nonanoic acid		-	0.81 ± 0.01 ^f^	1.06 ± 0.02 ^e^	-	1.37 ± 0.02 ^d^	-
2-Hexenoic		-	-	-	2.11 ± 0.01 ^d^	-	-
Subtotal		0	3.20 ± 0.21 ^a^	1.06 ± 0.02 ^e^	2.68 ± 0.04 ^b^	1.37 ± 0.02 ^e^	0
Aldehydes							
Pentanal		1.05 ± 0.02 ^f^	-	-	6.76 ± 0.15 ^a^	-	1.13 ± 0.12 ^f^
Heptanal	Herb	-	4.87 ± 0.32 ^fg^	15.07 ± 0.36 ^b^	0.51 ± 0.01 ^j^	15.52 ± 0.07 ^b^	0.25 ± 0.03 ^j^
Octanal		-	0.66 ± 0.02 ^d^	-	-	-	-
Nonanal		-	-	2.73 ± 0.05 ^e^	4.77 ± 0.52 ^b^	-	0.63 ± 0.08
Decanal	Citrus Peel	2.51 ± 0.03 ^c^	-	1.99 ± 0.12 ^d^	-	2.07 ± 0.12 ^d^	-
Benzaldehyde		-	3.45 ± 0.16 ^fh^	8.74 ± 1.10 ^c^	2.94 ± 0.06 ^i^	6.25 ± 0.61 ^de^	4.27 ± 0.30 ^f^
Phenylacetaldehyde	Hyacinth	1.59 ± 0.02 ^e^	1.05 ± 0.33 ^ef^	-	4.19 ± 0.31 ^c^	-	-
(Z)-2-hepental		8.51 ± 0.17 ^a^	0.21 ± 0.01 ^j^	2.92 ± 0.13 ^e^	-	3.23 ± 0.02 ^e^	0.61 ± 0.04 ^i^
(E)-2-nonenal		1.65 ± 0.08 ^e^	5.65 ± 0.31 ^b^	-	4.68 ± 0.22 ^c^	0.31 ± 0.02 ^j^	-
Subtotal		15.31 ± 1.46 ^i^	15.89 ± 1.22 ^i^	31.59 ± 3.24 ^a^	23.85 ± 2.45 ^d^	21.90 ± 1.97 ^e^	6.41 ± 0.41 ^k^
**Terpenes**							
α-Pinene	Resin	-	11.07 ± 0.26 ^f^	16.98 ± 1.05 ^c^	5.97 ± 0.14 ^i^	10.29 ± 1.22 ^f^	1.93 ± 0.04 ^i^
β-Pinene	Resin	5.72 ± 0.64 ^f^	18.08 ± 0.13 ^a^	-	7.19 ± 0.25 ^e^	8.26 ± 0.38 ^e^	-
Eucalyptol		25.18 ± 3.19 ^a^	15.38 ± 0.25 ^c^	4.13 ± 0.05 ^gf^	3.44 ± 0.09 ^g^	5.64 ± 1.04 ^f^	2.25 ± 0.21 ^h^
Nerol ooxide		9.14 ± 1.30 ^b^	-	4.34 ± 0.12 ^e^	-	7.65 ± 0.01 ^c^	1.30 ± 0.02 ^g^
Linalool		139.53 ± 0.15 ^e^	10.04 ± 1.05 ^h^	19.82 ± 1.30 ^f^	-	20.13 ± 0.22 ^f^	-
Neral	Grass	11.78 ± 1.36 ^de^	17.51 ± 1.11 ^c^	-	-	-	5.48 ± 0.06 ^h^
Geranial cis-Linalool oxide	Rose	10.86 ± 0.24 ^f^	1.44 ± 0.03 ^j^	6.72 ± 0.20 ^h^	17.67 ± 1.32 ^d^	7.03 ± 0.06 ^g^	1.91 ± 0.01 ^j^
Citral Linalool oxide		6.72 ± 0.15 ^h^	14.50 ± 1.06 ^c^	10.04 ± 0.49 ^d^	15.39 ± 0.24 ^b^	10.85 ± 0.31 ^d^	8.71 ± 0.15 ^g^
Citronellol		19.25 ± 0.34 ^h^	615.77 ± 5.72 ^a^	-	71.63 ± 3.79 ^e^	5.75 ± 0.03 ^i^	106.37 ± 2.31 ^c^
Geranic acid	Vegetables	262.30 ± 6.97 ^a^	10.58 ± 0.04 ^j^	80.85 ± 5.37 ^c^	18.13 ± 1.00 ^i^	91.16 ± 2.35 ^b^	55.41 ± 3.06 ^g^
α-Phellandrene		7.54 ± 0.30 ^d^	6.19 ± 0.62 ^e^	4.13 ± 0.04 ^j^	8.84 ± 0.06 ^c^	-	-
α-Terpinene		34.84 ± 2.16 ^a^	4.98 ± 0.71 ^g^	10.80 ± 1.21 ^e^	3.06 ± 0.12 ^h^	11.21 ± 1.07 ^e^	-
α-Terpineol	Flower	-	3.73 ± 0.45 ^f^	3.91 ± 0.03 ^f^	0.89 ± 0.02 ^g^	-	10.83 ± 0.64 ^c^
β-Myrcene	Grass	3.34 ± 0.05 ^j^	12.26 ± 0.82 ^e^	-	3.91 ± 0.21 ^j^	19.97 ± 1.20 ^d^	13.65 ± 1.28 ^e^
β-Ocimene		24.50 ± 1.33 ^b^	7.64 ± 0.93 ^g^	18.92 ± 1.36 ^c^	-	11.23 ± 0.39 ^f^	7.26 ± 0.06 ^g^
(Z)-Limonene		-	4.79 ± 0.80 ^g^	-	14.20 ± 1.30 ^d^	-	-
γ-Terpinene	Lemon	34.03 ± 0.62 ^a^	1.41 ± 0.03 ^i^	-	-	9.49 ± 0.23 ^e^	7.43 ± 0.67 ^f^
4-Terpinene		11.63 ± 1.50 ^c^	-	-	0.21 ± 0.01 ^g^	-	0.95 ± 0.03 ^g^
o-Cymene		13.26 ± 0.06 ^d^	-	4.31 ± 0.45 ^j^	11.49 ± 1.04 ^d^	7.62 ± 0.26 ^h^	6.80 ± 0.11 ^i^
m-Cymene		-	1.04 ± 0.02 ^g^	-	3.53 ± 0.03 ^e^	-	-
Terpinolene	Nutty	24.96 ± 2.08 ^a^	-	1.87 ± 0.38 ^h^	4.21 ± 0.55 ^g^	2.18 ± 0.17 ^h^	-
Eugenol	Lilac	0.47 ± 0.02 ^h^	8.02 ± 0.30 ^d^	-	12.52 ± 1.08 ^c^	-	-
Hotrienol		36.66 ± 1.59 ^a^	16.45 ± 1.11 ^d^	3.11 ± 0.22 ^h^	-	4.82 ± 0.23 ^g^	13.51 ± 0.16 ^d^
Myrtenol	Mint	-	-	12.58 ± 0.01 ^f^	27.28 ± 1.21 ^b^	14.89 ± 0.35 ^e^	20.63 ± 1.22 ^c^
Isogeraniol		8.94 ± 0.05 ^f^	18.21 ± 0.57 ^c^	-	-	-	-
E-Nerolidol		6.73 ± 0.12 ^f^	12.13 ± 0.84 ^c^	5.35 ± 0.16 ^g^	14.29 ± 0.40 ^b^	9.66 ± 0.50 ^e^	0.95 ± 0.07 ^k^
cis-β-Ocimene		15.40 ± 0.06 ^b^	-	-	3.54 ± 0.06 ^f^	-	8.42 ± 0.31 ^d^
trans-β-Ocimene	Citrus	20.06 ± 0.21 ^a^	-	17.26 ± 1.07 ^b^	13.05 ± 0.89 ^e^	-	12.39 ± 1.10 ^e^
cis-Rose oxide	Rose	31.51 ± 2.13 ^a^	17.15 ± 1.62 ^c^	-	16.91 ± 1.73 ^cd^	-	-
trans-Rose oxide	Litchi	13.30 ± 0.66 ^e^	28.91 ± 2.15 ^a^	19.24 ± 1.26 ^c^	-	19.55 ± 0.34 ^c^	11.62 ± 0.51 ^f^
Subtotal		719.69 ± 15.41 ^d^	879.50 ± 22.37 ^c^	301.14 ± 10.55 ^h^	225.89 ± 5.16 ^i^	421.45 ± 8.19 ^g^	253.25 ± 5.21 ^hi^
**C13-Norisoprenoids**							
β-Damascenone	Honey	1.86 ± 0.03 ^a^	1.07 ± 0.02 ^b^	-	0.62 ± 0.10 ^f^	-	-
β-Ionone	Violets	1.43 ± 0.15 ^ab^	1.65 ± 0.08 ^a^	0.47 ± 0.05 ^e^	-	0.78 ± 0.01 ^cd^	0.91 ± 0.04 ^c^
Geranylacetone	Flower	1.10 ± 0.02 ^c^	1.41 ± 0.11 ^b^	-	-	-	0.27 ± 0.02 ^f^
Subtotal		3.43 ± 0.21 ^c^	4.63 ± 0.30 ^a^	0.47 ± 0.05 ^fg^	0.62 ± 0.10 ^ef^	0.96 ± 0.01 ^e^	1.18 ± 0.07 ^d^
TOTAL		4150.27 ± 23.46 ^h^	6566.03 ± 57.19 ^c^	2558.30 ± 26.20 ^k^	2046.64 ± 18.85 ^kl^	3121.05 ± 30.02 ^i^	2719.55 ± 38.41 ^k^

Note: different lowercase letters in the same row indicate significant differences (*p* < 0.05); ‘-’ means the substance was not detected.

## Data Availability

The related data and methods are presented in this paper. Additional inquiries should be addressed to the corresponding author.

## Supplementary Materials

The following supporting information can be downloaded at: https://www.mdpi.com/article/10.3390/foods12183371/s1, Table S1: Introduction of tested grape varieties; Table S2: Sensory evaluation criteria for table grapes.
