# Supplementary material for: Effects of Table Grape Cultivars on Fruit Quality and Aroma Components
Source: Foods. 2023 Sep 8;12(18):3371. doi: 10.3390/foods12183371 (PMC10530201; doi:10.3390/foods12183371)
Supplement: Supplementary file 1 [file foods-12-03371-s001.zip › foods-2595470-supplementary.pdf]

Table S1 Introduction of tested grape varieties

Number	Name of species	Type of species	Location of test field	Sampling time	color
A	*Heibaladuo*	*Vitis vinifera* L.	B-24-S15	2021.8.1	red
B	*Meixiangbao*	Franco-american	B-22-S15	2021.8.4	red
C	*Italy*	*Vitis vinifera* L.	B-3，4	2021.8.6	white
D	*Sunmmer Black*	Franco-american	B-25-M16	2021.8.7	red
E	*Shaoxing 1 hao*	*Vitis vinifera* L.	B-20-N16	2021.8.8	red
F	*Zaoheibao*	*Vitis vinifera* L.	B-25-S15	2021.8.10	red
G	*Manicure Finger*	*Vitis vinifera* L.	B-9，10	2021.8.13	red
H	*Aishenmeigui*	*Vitis vinifera* L.	B-22-N16	2021.8.19	red
I	*Zhengyanwuhe*	*Vitis vinifera* L.	B-18-N16	2021.8.22	red
J	*Sweet Sapphire*	*Vitis vinifera* L.	B-23-S15	2021.8.23	red
K	*Ruby Seedless*	*Vitis vinifera* L.	B-19-M16	2021.8.28	red
L	*Ruiduwuheyi*	*Vitis vinifera* L.	B-18-S15	2021.8.29	red
M	*Jumeigui*	Franco-american	B-1，2	2021.9.7	red
N	*Shine-Muscat*	Franco-american	B-7，8	2021.9.16	white
O	*Hutai 8 hao*	Franco-american	B-11，12	2021.9.16	red
P	*Heisetiancai*	Franco-american	B-25-N16	2021.9.22	red
Q	*Zitianwuhe*	*Vitis vinifera* L.	B-22-M16	2021.9.24	red

Table S2 Sensory evaluation criteria for table grapes

Project (100)		Scoring Range	Score
Appearance (30)	Ear drop, loose, stem withered, poor fruit shape, fruit wilt, juice overflow, no fruit powder	1-10	
	The ear was compact and moderate, the peduncles were brown, the fruit color was dark, and the fruit powder dropped off a little	10-20	
	The ear is compact, the stem is fresh and tender, the fruit shape is correct, the fruit color is bright, the fruit powder is full	20-30	
Texture (30)	The pericarp is rough, the pulp is soft, and the pulp and pericarp are separated in bags	1-10	
	The skin is not tough enough, and the flesh is soft and brittle	10-20	
	The skin is puffed and cracked easily, and the flesh is thick, juicy and rich in texture	20-30	
Flavor (40)	The flavor is uncoordinated, the acidity is too big, the astringent taste is slightly heavy, has the bad smell	1-15	
	Good flavor, less sweet, no bad smell	15-30	
	Excellent flavor, harmonious sugar-acid ratio, grape-specific aroma rich	30-40	
